# Mesoporous silica/organosilica nanoparticles for cancer immunotherapy

**DOI:** 10.1002/EXP.20220086

**Published:** 2023-05-09

**Authors:** Shevanuja Theivendran, Sergei Lazarev, Chengzhong Yu

**Affiliations:** ^1^ Australian Institute for Bioengineering and Nanotechnology The University of Queensland, Brisbane St Lucia Australia

**Keywords:** cancer, immunotherapy, nanoparticles, organosilica, silica

## Abstract

Cancer is one of the fatal diseases in the history of humankind. In this regard, cancer immunotherapeutic strategies have revolutionized the traditional mode of cancer treatment. Silica based nano‐platforms have been extensively applied in nanomedicine including cancer immunotherapy. Mesoporous silica nanoparticles (MSN) and mesoporous organosilica nanoparticles (MON) are attractive candidates due to the ease in controlling the structural parameters as needed for the targeted immunotherapeutic applications. Especially, the MON provide an additional advantage of controlling the composition and modulating the biological functions to actively synergize with other immunotherapeutic strategies. In this review, the applications of MSN, MON, and metal‐doped MSN/MON in the field of cancer immunotherapy and tumor microenvironment regulation are comprehensively summarized by highlighting the structural and compositional attributes of the silica‐based nanoplatforms.

## Graphical abstract

Silica based nanoparticles have been extensively applied in nanomedicine including cancer immunotherapy. In this review, the applications of mesoporous silica nanoparticles (MSN), mesoporous organosilica nanoparticles (MON), and metal‐doped MSN/MON in the field of cancer immunotherapy and tumor microenvironment regulation are comprehensively summarized by highlighting the structural and compositional attributes of the silica‐based nanoplatforms.

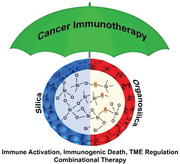

## 1 INTRODUCTION

Since the discovery of ordered mesoporous silicate materials in 1992,^[^ ^1^ ^]^ surfactant templated formation of silica structures has gained huge interest among material scientists. This assembly strategy allows for a high level of structural control with respect to the specific surface area, pore sizes, and pore volumes. Shaping the morphology of mesoporous silicate materials into nanosizes led to the development of mesoporous silica nanoparticles (MSN) with broad applications such as catalysis, adsorption, ion exchange, and sensing.^[^ ^2^, ^3^, ^4^, ^5^ ^]^ In 2001, Pérez‐Pariente et al. discovered the interesting property of MSN as a drug delivery system.^[^ ^6^ ^]^ Following this, MSN have been used as excellent drug carriers and applied for different therapeutic applications, including cancer therapy, immunotherapy, tumor microenvironment modulation, tissue engineering, anti‐infective therapy, and diabetes treatments.^[^ ^7^, ^8^, ^9^, ^10^, ^11^, ^12^ ^]^ Despite their advantages, the plain chemistry of bare silica and the poor bio‐degradability of silica framework have limited the wide biomedical applications of MSN.^[^ ^13^, ^14^ ^]^

In 1999, periodic mesoporous organosilicas with organic groups uniformly distributed in the inorganic silica framework were reported.^[^ ^15^, ^16^, ^17^ ^]^ Incorporation of both organic and inorganic components has opened new opportunities in adjusting the structural, physiochemical, and biocompatible properties of ordered mesoporous materials.^[^ ^18^ ^]^ Since then, hybrid mesoporous organosilica materials with various organic groups especially in nanoparticle forms have been reported.^[^ ^18^ ^]^ Mesoporous organosilica nanoparticles (MON) can be categorized mainly into three types. 1) Nanoparticles purely made from organosilica precursors, without any inorganic silica precursors. 2) Nanoparticles obtained by the post modification of preformed MSN/MON with organosilane. 3) Nanoparticles prepared by incorporating both the organic and inorganic silica precursors in the framework. Organosilicas synthesized with pure organosilane precursors have shown improved loading/binding performance toward various hydrophobic drugs.^[^ ^19^ ^]^ Surface grafting of organosilane groups allowed for further modification of different entities such as drug molecules, fluorescent dyes, and chemical moieties.^[^ ^20^, ^21^, ^22^, ^23^ ^]^ Furthermore, the incorporation of organosillica groups into the inorganic silica framework improved the functional characteristics such as biodegradability, colloidal stability, and the stimuli responsive release of cargo from the nanoparticles, making them more suitable for bioapplications compared to MSN with a bare silica composition.^[^ ^18^, ^24^, ^25^ ^]^

Cancer immunotherapy has revolutionized the traditional modes of cancer treatments due to the advantage of treating cancer without direct killing of cancer cells by chemo drugs and reduced side effects compared to conventional cancer treatment methods such as radiation therapy and chemotherapy.^[^ ^26^ ^]^ Immunotherapeutic methods trigger the innate immune system of the patients to identify and combat cancer.^[^ ^27^ ^]^ In recent years, there is also an emerging interest in understanding and targeting various components of tumor microenvironment such as tumor vasculature, tumor associated macrophages, T cells, dendritic cells (DC), stroma, and other physiochemical changes in tumor to enhance the therapeutic outcome by alleviating the immune suppressive and resistive nature of cancer.^[^ ^28^, ^29^, ^30^ ^]^ Immune checkpoint blockade (ICB) therapy is a well‐known cancer immunotherapy and Nobel prize has been awarded to the pioneers in this field in 2018.^[^ ^31^ ^]^ In recent years, silica based nanoparticles have also been applied for cancer immunotherapy to improve the efficacy and safety of different immunotherapeutic strategies.

Various synthetic strategies have been developed for controlling the size, composition, shape, porous structure, and dispersity of MSN/MON to produce suitable candidates for nano‐biomedicine. Owing to the unique compositional and physiochemical characteristics, MON have been widely applied in many biomedical applications in recent years, including cancer therapy,^[^ ^25^ ^]^ theranostics,^[^ ^24^ ^]^ and immunotherapy,^[^ ^32^ ^]^ and have illustrated improved functions and performance than MSN in many cases. For example, the incorporation of tetrasulfide bridging group into the framework greatly improved the degradable nature of silica nanoparticles and attracted huge attention for the cargo delivery in glutathione (GSH) rich cancer cells in a stimuli responsive manner.^[^ ^33^ ^]^ In addition to the preparation of whole degradable nanoparticles, degradable organosilica can be used as a gating material for controlled release of payload.^[^ ^34^, ^35^ ^]^ Such a design avoids the premature release of cargo, which can potentially improve the efficacy of nanoformulations and reduce possible side effects.

The addition of different kinds of metal species such as Fe, Cu, and Mn has also elevated the therapeutic potential of silica as well as the organosillica nanoparticles.^[^ ^36^, ^37^, ^38^ ^]^ The metal components can be either decorated or incorporated into silica/organosilica framework, or loaded into the pores of silica/organosilica components. Considering the biological anomalies that exist within the cancer cells and/or the tumor microenvironment, such as the hypoxia, acidic pH, and GSH level, specific metals can be incorporated in the formulation to achieve stimuli‐responsive biofunctions and improved therapeutic outcomes.^[^ ^39^, ^40^, ^41^ ^]^

**SCHEME 1:**
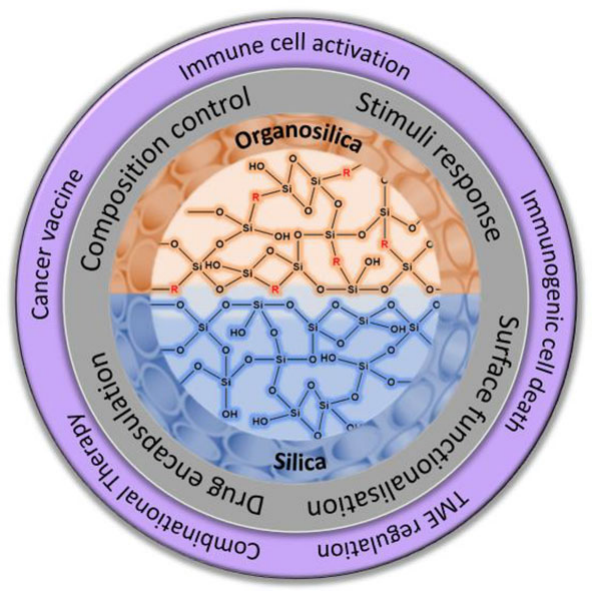
Schematic illustration on the attributes of silica and organosilica based nanoparticles and their application in different branches of cancer immunotherapy.

To date, there are several excellent reviews on the synthesis, morphology, and framework control of MSN or MON, functional difference between silica and organosilica nanoparticles, and organosilica nanoparticles in therapeutic and theranostic applications.^[^ ^11^, ^18^, ^24^, ^25^, ^42^ ^]^ However, to the best of our knowledge, a dedicated review focusing specifically on MSN, MON, and metal‐doped MSN/MON for cancer immunotherapy and tumor microenvironment (TME) modulation is rare. This review aims to provide a summary of the recent research progress in this direction. As illustrated in Scheme 1, this review focuses on summarizing the use of MSN and MON with unique characteristics and functionalities for different cancer immunotherapeutic strategies. First, we will discuss about the design of innovative silica and organosilica nanostructures with unique pore size, structural control, and compositional architecture. Second, we will move on to the advantages of nanoparticles with engineered structural elements and functions for therapeutic applications in cancer immunotherapy and TME modulation. Third, the biocompatibility assessment of MSN and MON in the interest of in vivo applications of such nanoplatforms will be reviewed. Finally, an outlook of challenges and prospects of silica‐based nanomedicine for future cancer immunotherapy and clinal translation will be discussed.

## 2 SYNTHESIS OF MSN AND MON

The synthesis of MSN and MON with controllable structures has been extensively reviewed. The readers are referred to the latest review articles for comprehensive and systematic information.^[^ ^42^, ^43^, ^44^ ^]^ In this section we will provide a concise summary on the key difference between MSN and MON in their synthesis, general principles of pore size, and morphology control. The composition of MSN and MON is dependent on the types of silica precursors used in the synthesis, which undergo hydrolysis and condensation during the reaction.^[^ ^45^ ^]^ In the presence of catalysts (e.g., OH^−^), the generated silanol groups from the hydrolysis of the silane precursors (e.g., ethoxysilane for pure silica and bissilylated organoalkoxysilane for organosilica) will further condense to form siloxane bonds. The assembly between surfactant templates and partially hydrolyzed/condensed species leads to the formation of MSN or MON. For example, the use of only bissylated organosilica precursors leads to the formation of MON with a pure organosilica composition.^[^ ^42^ ^]^ However, the products made from mixed pure silica and organosilica precursors, including bissilylated ones or with an end R group (e.g., R is an alkyl group) through either co‐condensation or post‐modification, are also named as MON in literature. It is pointed out that the hydrolysis and condensation rates of the bissilylated organosilica precursors vary at different synthesis pH and differ significantly from inorganic silica precursors. Thus, the synthesis of MON with high organic contents and controllable structures is relatively difficult compared to that of MSN.

Nanoparticles with large pore sizes while maintaining small particle sizes (less than 100 nm) are preferred for the delivery of biomolecules with high molecular weights for therapeutic applications. Structure directing agents such as cetyltrimethylammonium bromide (CTAB) and various micelle swelling agents have been used to enlarge the pore size of MSN/MON for large biomolecule delivery applications.^[^ ^46^, ^47^, ^48^, ^49^, ^50^ ^]^ Furthermore, the pore size of the nanoparticles can be also enlarged by tuning the concentration of incoming precursor amounts or by introducing oil (pore swelling agent) into the reaction system, while keeping the nanoparticle size small.^[^ ^51^, ^52^ ^]^ For example, Zhang et al. reported the synthesis of magnetic MSN where CTAB was used as the template and 1,3,5‐triisopropylbenzene (TMB)/decane was used as the pore swelling agent. In this work, the nanoparticle size was maintained in the range of 40–70 nm while the pore size was enlarged up to 6.1 nm.^[^ ^53^ ^]^ In another study, Yang et al. reported the use of toluene as the swelling agent for the synthesis of benzene bridged organosilica nanoparticles with the particle size of 50 nm and pore size of 7 nm. In this case, the use of oil has dual functions of assisting surfactant/organosilica assembly and enlarging mesopores.^[^ ^54^ ^]^ In another report, Qin et al. reported the use of hydrophobic polystyrene as a pore‐swelling agent for the enlargement of MSN pore channels up to 30 nm. The authors further demonstrated that the pore hierarchy can be also tuned by changing the amount of polystyrene used in the reaction system.^[^ ^55^ ^]^

The morphology of MSN and MON can be also tuned simply by adjusting the cosolvents^[^ ^56^ ^]^ or surfactant ratios in the reaction mixture,^[^ ^57^ ^]^ stirring speed,^[^ ^58^ ^]^ and by changing the type of bissylated organosilica precursors with different bridging groups.^[^ ^59^, ^60^ ^]^ For example, Fatieiev et al. reported the use of different cosolvents such as toluene, cyclohexane, and dimethylformamide to dissolve the organosilica precursor to obtain periodic mesoporous organosilicas with different morphologies. The authors used 1,4‐*bis*(triethoxysilyl)benzene as the precursor and produced nanowires in toluene, nanorods in cyclohexane, bent nanorods in toluene/DMF cosolvent mixture, and nanospheres in DMF.^[^ ^56^ ^]^ MSN of different shapes such as rod‐like,^[^ ^61^ ^]^ worm‐like,^[^ ^62^, ^63^ ^]^ dendritic,^[^ ^64^ ^]^ concentric,^[^ ^65^ ^]^ foam‐like^[^ ^65^ ^]^ and cubic^[^ ^66^ ^]^ have been reported, to name a few. Zhao et al. prepared MSN with different aspect ratios by simply tuning the amount of ammonia used in the synthesis process.^[^ ^66^ ^]^ Similarly, Yu et al. tuned the aspect ratio of MON simply by changing the CTAB to ammonia ratio to obtain rod‐like MON with different length‐diameter ratios.^[^ ^57^ ^]^

Over the years, various hybrid MON with different organic compositions, such as methylene, ethylene, *bis*(propyl)disulfide, *bis*(propyl)tetrasulfide, phenylene, and biphenylene as bridge groups, have been prepared.^[^ ^59^, ^67^ ^]^ Incorporation of organic bridging groups improved the functional characteristics and biodegradability of nanoparticles. Mai et al. reported the use of benzene and tetrasulfide bridge group incorporated organosilica nanoparticles for the controlled and slow release of a chemo drug cordycepin. The aromatic rings in the cordycepin exhibited strong interaction with the benzene rings in the silica framework.^[^ ^68^ ^]^ Fan et al. synthesized hollow organosilica nanoparticles with multiple organic bridging groups for the co‐delivery of drugs for the synergistic radiodynamic and gas therapy.^[^ ^69^ ^]^

In this regard, silica based nanoplatforms are one of the widely used nanoparticle types for biomedical applications especially for cancer treatment. MSN is an attractive cargo carrier, whereas MON revolutionized the use of silica nanoplatforms as not just carriers but biomodulators in various biomedical applications.^[^ ^70^ ^]^ The applications of MSN and MON in cancer immunotherapy including tumor microenvironment modulation, cancer vaccine, and gene delivery will be discussed in detail in the following sections. A summary of the application of MSN/MON and metal‐doped MSN/MON for different cancer immunotherapy applications is also presented in Table 1.

**TABLE 1:** A table summarizing the application of MSN/MON and metal‐doped MSN/MON for different cancer immunotherapy applications.

Nanoparticle type	Composition	Net size (nm)	Pore size (nm)	Surface area	Application	Property	Ref.
3.1 MSN for drug mediated immunotherapy							
MSN	–	150–270	4	–	Doxorubicin delivery	ICD Enhanced cellular uptake	[75]
MSN	–	80–100	–	–	TME regulation Indoximod and oxaliplatin delivery	ICD Reverse of immunosuppression	[76]
MSN	–	50–100	–	–	Doxorubicin, 1‐methyltryptophan delivery	Controlled degradation/release DC and T cell activation	[77]
MSN	–	100–180	–	–	Doxorubicin delivery	ICD High tumor accumulation Lipid encapsulation provides better results	[78]
MSN	Au/Mn	105–120	3	267 m^2^ g^−1^	TME regulation Doxorubicin and aspirin delivery	ICD Hydroxyl radical generation	[79]
MSN	–	200	2–4	1066 m^2^ g^−1^	Doxorubicin delivery, CTLA‐4 combinational therapy	ICD Tumor growth inhibition Enhanced macrophage and dendritic cell tumor accumulation	[80]
MSN	–	160–300	2–3	71 m^2^ g^−1^	Doxorubicin delivery Enhanced with RGD and laser combinational therapy	ICD Targeting RGD peptide incorporation	[81]
MSN	Au	110	–	–	TME regulation R837 adjuvant delivery Photo, immuno, and starvation combinational therapy	ICD Adjuvant delivery	[82]
MSN	As	100–160	1–3	–	Chlorin e6 delivery Photodynamic therapy	ICD NK activation High tumor accumulation in vivo	[85]
MSN	–	30–70|250–400	–	–	Chlorin e6, PTX delivery Anti‐PD‐L1 antibody combinational therapy	ICD Metastasis prevention Intratumoral immune system activation	[86]
3.2 MSN as a delivery platform for cancer vaccine, antigens, and adjuvants							
MSN	–	80	–	–	STING, CDA delivery for tumor inhibition	Biodegradation Dendritic cell activation Innate and adaptive immune cell activation	[88]
MSN	–	150	10–30	342 m^2^ g^−1^	Antigen, antibody delivery	DC activation	[89]
MSN	–	300–380	3	–	TME regulation Adjuvant delivery Gardiquimod delivery Photothermally activated nanoparticles, combinational therapy	Biodegradation NIR activation Tumor associated antigen generation	[90]
MSN	–	250	–	–	OVA delivery Combinational photo/immunotherapy Antigen delivery	Photothermally activated nanoparticles Melanoma eradication rate of 75%.	[91]
MSN	CuS	100–150	–	–	Combinational photo/immunotherapy Immune adjuvant resiquimod R848 delivery	Photothermal activation Incorporated anti‐PD‐1 targeting ligand	[92]
MSN	Ca/Mg/Zn	100	4	–	Cancer adjuvant delivery	Biodegradation Th1 inducing effect	[93]
MSN	–	200	3–4	235 m^2^ g^−1^	Cancer vaccine adjuvant PEI incorporation	Biodegradation Dendritic cell induction Significant reduction of metastasis	[94]
MSN	Ti	280	34	250–400 m^2^ g^−1^	Doxorubicin delivery	Significant cancer growth inhibition	[95]
MSN	Fe‐Ru	100–150	–	–	TME regulation Induce tumor specific mitochondrial DNA damage	Macrophage polarization	[98]
3.3 MSN as gene delivery vehicle							
MSN	–	200	2–6	990–1020 m^2^ g^−1^	Adjuvant OVA delivery	Increased anti‐cancer immunity	[100]
MSN	–	100–200	> 35	250–560 m^2^ g^−1^	OVA adjuvant, gene dsRNA delivery	Anti‐cancer immunity	[101]
3.4 MSN for TME modulation							
MSN	–	186–243	–	660–940 m^2^ g^−1^	ATRA, doxorubicin, IL‐2 delivery	TME regulation Immune cell activation Biodegradation	[96]
DMSN	–	68–170	–	300–480 m^2^ g^−1^	miRNA delivery for cancer immunotherapy TME regulation	Increased cellular uptake GSH induced degradation release	[97]
DMSN	CaO_2_/Fe_3_O_4_	110	–	–	TME regulation Tumor growth inhibition Tumor associated macrophage polarization	ICD pH responsive release Hydroxyl radical generation	[99]
3.5 MON for drug delivery							
DMON	Cu/─S─S─	210	10–20	–	Doxorubicin delivery	ICD Controlled release and degradation Immunogenic response activation	[36]
MON	Fe_3_O_4_/─S─S─	250 × 100	4	720 m^2^ g^−1^	TME regulation Chlorine e6 delivery Combinational chemo/hyperthermia therapy	ICD Redox/pH activatation Biodegradation ROS augmentation	[102]
MON	─S─S─	165	21	460 m^2^ g^−1^	Curcumin delivery	ICD Biodegradation Hydroxyl radical generation	[103]
MON	Fe/─S─S─	360	6	890 m^2^ g^−1^	Adjuvant OVA delivery TME regulation	ICD T cell/macrophage polarization Dendritic cell activation	[37]
MON	─Se─Se─	60	4	430 m^2^ g^−1^	Doxorubicin delivery TME regulation	ICD X‐ray responsive degradation	[32]
MON	─Se─Se─	65	5–7	530 m^2^ g^−1^	TME regulation KP1339 delivery Antitumor immunity amplification	ICD Biodegradation	[104]
MON	─Se─Se─	60–80	5–7	730 m^2^ g^−1^	Doxorubicin and methylene blue delivery	ICD Phototherapy activation Immunogenic response Metastasis suppression	[105]
3.6 MON for cancer vaccine for the delivery of antigens and adjuvants							
MON	─C─C─	200–530	14–18	300–360 m^2^ g^−1^	Adjuvant OVA delivery	Adjuvant delivery	[106]
MON	─S─S─	200	10–30	317 m^2^ g^−1^	Adjuvant OVA, TLR9 delivery	GSH induced biodegradation Enhanced anti‐tumor immunity	[107]
MON	Benzene	110–144	8–16	540–800 m^2^ g^−1^	Adjuvant OVA, TLR9 delivery	Inhibition of B16 melanoma	[108]
3.7 MON for TME modulation							
HMON	─S─S─	180–220	4	635 m^2^ g^−1^	TME regulation Hydroxycamptothecin delivery	Lung metastasis inhibition Tumor associated macrophage polarization	[109]
DMON	─S─S─	230	45–46	250–390 m^2^ g^−1^	Nitric oxide delivery	Biodegradation M1 phenotype macrophage polarization	[110]
3.8 MON for gene delivery							
HMON	Benzene	450–550	2–25	520 m^2^ g^−1^	Doxorubicin, siRNA delivery	Biodegradation Anti tumor effect	[111]
MON	─S─S─	70	4–9	440 m^2^ g^−1^	Doxorubicin, siRNA delivery	ICD Tumor growth inhibition	[112]

Abbreviations: DMON, dendritic mesoporous organosilica nanoparticles; ICD, immunogenic cell death; MON, mesoporous organosilica nanoparticles; MSN, mesoporous silica nanoparticles; OVA, ovalbumin; STING, STimulator of INterferon genes; TME, tumor microenvironment.

## 3 CANCER IMMUNOTHERAPY

Cancer immunotherapy has emerged as a powerful therapeutic strategy that has the potential to enhance therapeutic outcome while reducing the side effects of conventional cancer treatments. Fundamentally, cancer immunotherapy relies on the patient's immune system itself to evoke immune response against cancer cells.^[^ ^26^ ^]^ Over the recent years, there has been an exponential increase in the research on cancer immunotherapy and various new techniques such as ICB and chimeric antigen receptor (CAR) T cell therapy have been discovered.^[^ ^26^ ^]^ However, the clinical success is still limited due to the challenges in efficacy, safety, and cost‐effectiveness.^[^ ^31^ ^]^

Novel delivery strategies have been discovered to improve the efficacy of immunotherapy through targeted delivery and reduction in off‐target effects to overcome safety concerns.^[^ ^26^ ^]^ Nanoparticles such as silica, lipid, metal, and polymer nanoparticles have been explored for this purpose.^[^ ^31^ ^]^ Compared to other nano delivery systems, silica‐based nano‐systems have their own advantages. The silica nanoparticles have simple synthetic processes and easily tunable structural properties such as size, shape, pore size, and specific surface area compared with other drug delivery systems. The tunable pore sizes of 2–50 nm, large specific surface areas (> 1000 m^2^ g^−1^), and ease of surface modification are advantageous for delivery of various therapeutic agents such as drugs, antigens, adjuvants, proteins, and genes with different sizes and properties.^[^ ^13^ ^]^ Moreover, successful loading and release of cargo materials for cancer immunotherapy also depends on the choice of apt pore size of the nanoparticles. Hong et al. demonstrated that, in the case of antigen delivery, the antigen cross‐presentation can be tuned by adjusting the pore sizes of MSN. The authors concluded that the MSN with a large pore size (∼ 13 nm) induced significant anti‐tumor effect and cellular immune responses compared to smaller pore sizes (∼ 8 and ∼ 10 nm).^[^ ^71^ ^]^ In addition, the nanoparticles with small pore sizes can be used for the delivery of small molecular weight drugs whereas the nanoparticles with large pore sizes are often required for the delivery of macromolecules such as proteins, antigens, adjuvants, immune stimulatory molecules, and genetic material for successful immunotherapy.^[^ ^72^, ^73^ ^]^ Apart from the structural advantages of MSN for therapeutic applications, the possibility of framework control of nanoparticles further advances the responsive nature of nanoparticles in different biological cues. For example, MON with tetrasulfide bridged framework is applied for the responsive release of cargo in GSH rich cell types which minimizes the off‐target effects compared to MSN.^[^ ^24^ ^]^

### 3.1 MSN for drug‐mediated immunotherapy

**FIGURE 1:**
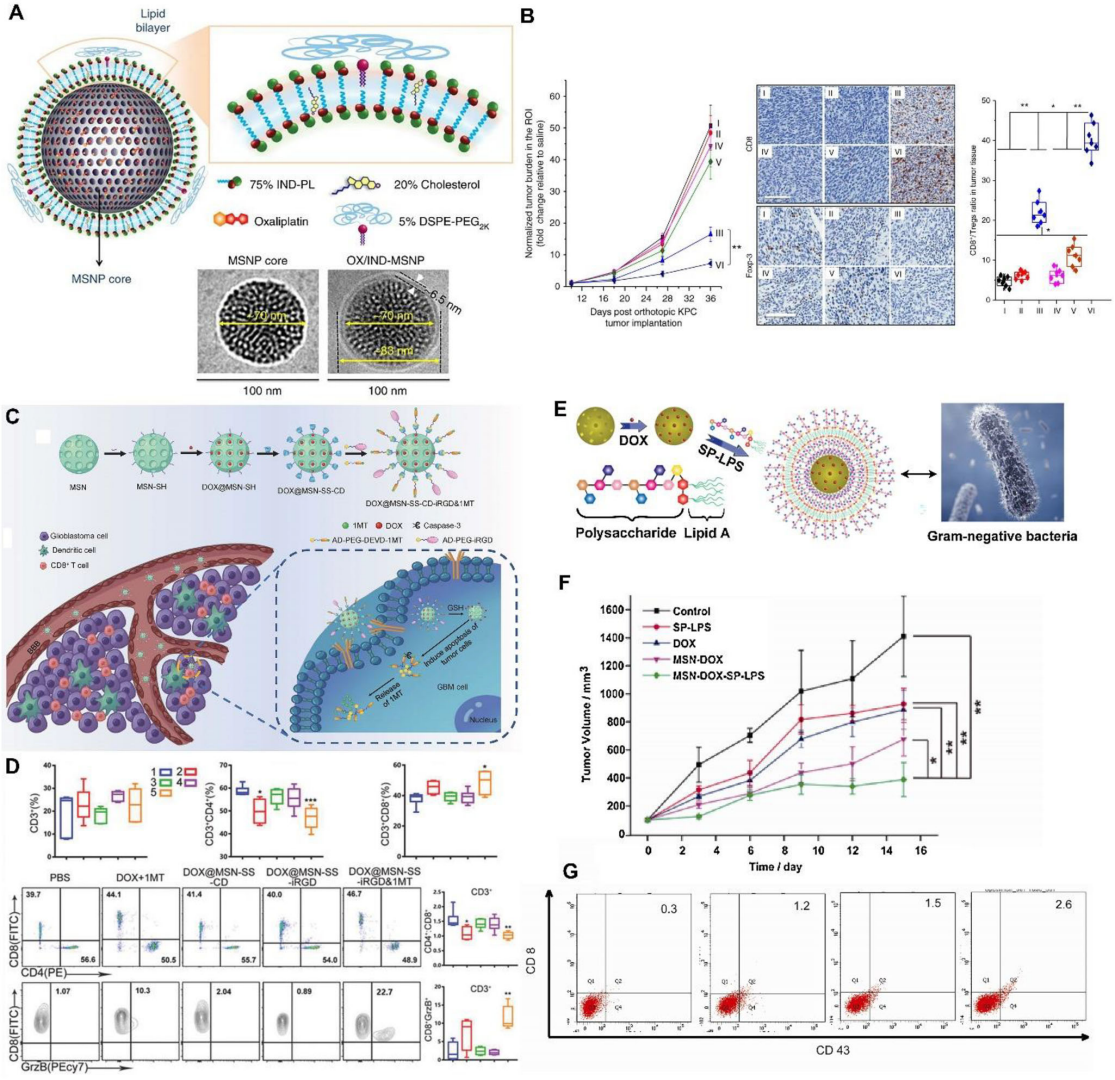
Mesoporous silica nanoparticles (MSN) for drug delivery applications. Schematic illustration of the A) indoximod phospholipid prodrug (IND‐PL) nanoformulation and B) its anti‐tumor effects in tumor tissue. Reproduced with permission.^[^ ^76^ ^]^ Copyright 2017, Nature Portfolio. Schematic illustration of the C) modification process of MSN for treating Glioblastoma and D) its T cell activation efficiency in the tumor site. Reproduced with permission.^[^ ^77^ ^]^ Copyright 2018, Wiley. Schematic illustration of the E) pathogen mimicking nanoformulation and F,G) its tumor inhibition profile as well as T cell activation efficacy. Reproduced with permission.^[^ ^78^ ^]^ Copyright 2017, Elsevier.

Researchers applied MSN as a versatile drug carrier due to the feasibility of synthesizing nanostructures with high specific surface area and large pore sizes. Doxorubicin (Dox) and oxaliplatin are well known immunogenic cell death (ICD) inducing agents, which can induce cancer cell death and the subsequent immune response through the production of damage‐associated molecular patterns (DAMPS) and tumor associated antigens (TAA).^[^ ^74^ ^]^ Numerous studies have reported the use of MSN for the delivery of Dox in tumor and measured the subsequent induction of immune response. For instance, Xu et al. prepared a virus like hollow MSN loaded with Dox for treating 4T1 breast cancer. It was demonstrated that compared to hollow MSN, virus‐like MSN had a better anti‐tumor effect due to enhanced cellular uptake.^[^ ^75^ ^]^ Easy surface modification of MSN was further utilized for the post modification of tumor targeting ligands for targeted release of drugs. Lu et al. reported a prodrug nanoformulation using MSN with an average size of 70 nm loaded with Oxaliplatin and coated with lipid layer containing indoximod, an immunosuppressive metabolic enzyme (IDO) inhibitor (Figure 1A,B). The prodrug nanoformulation demonstrated a synergistic immunotherapy in pancreatic ductal adenocarcinoma through the successful induction of ICD and reversal of the immunosuppressive effects of IDO.^[^ ^76^ ^]^

Kuang et al. formulated a drug delivery system using MSN coloaded with Dox and 1‐methyltryptophan (an immune checkpoint inhibitor), which was further modified with a peptide that can penetrate the tumor tissue and target tumor cells (iRGD) for treating Glioblastoma (Figure 1C). This formulation resulted in better penetration through the blood brain barrier and elicited robust anti‐tumor immune response with higher population of DC and cytotoxic T cells recruited into the tumor region compared to the drug treatment group without nanoparticles (Figure 1D).^[^ ^77^ ^]^ As shown in Figure 1E, Dong et al. report the first pathogen mimicking MSN for chemo‐immunotherapy, where MSN was loaded with Dox and surface functionalized with detoxified lipopolysaccharides (LPS; mimics natural pathogen for triggering immune responses). In this work, LPS was linked to MSN using an arylboronic ester, which is oxidized in the presence of ROS for effective delivery of drug. The pathogen mimicking nanosystem exhibited potent tumor inhibition and T cell activation (Figure 1F,G).^[^ ^78^ ^]^ In another report, gold nanoparticles coated with hollow MSN‐doped with manganese and loaded with Dox and Aspirin were prepared for synergistic chemoimmunotherapy.^[^ ^79^ ^]^ The Fenton like reaction was mediated by Mn^2+^ whereas the H_2_O_2_ was produced by the glucose oxidase like reaction mediated by gold nanoparticles. This combinational chemodynamic therapy in conjunction with Dox induced enhanced ICD for the subsequent stimulation of anti‐tumor immunity. In a similar fashion, MSN has been loaded/coloaded with drugs or agents to induce synergistic responses through chemotherapy,^[^ ^80^ ^]^ photothermal therapy,^[^ ^81^, ^82^, ^83^, ^84^ ^]^ and photodynamic therapy.^[^ ^85^, ^86^, ^87^ ^]^

### 3.2 MSN for the delivery of antigens and adjuvants

**FIGURE 2:**
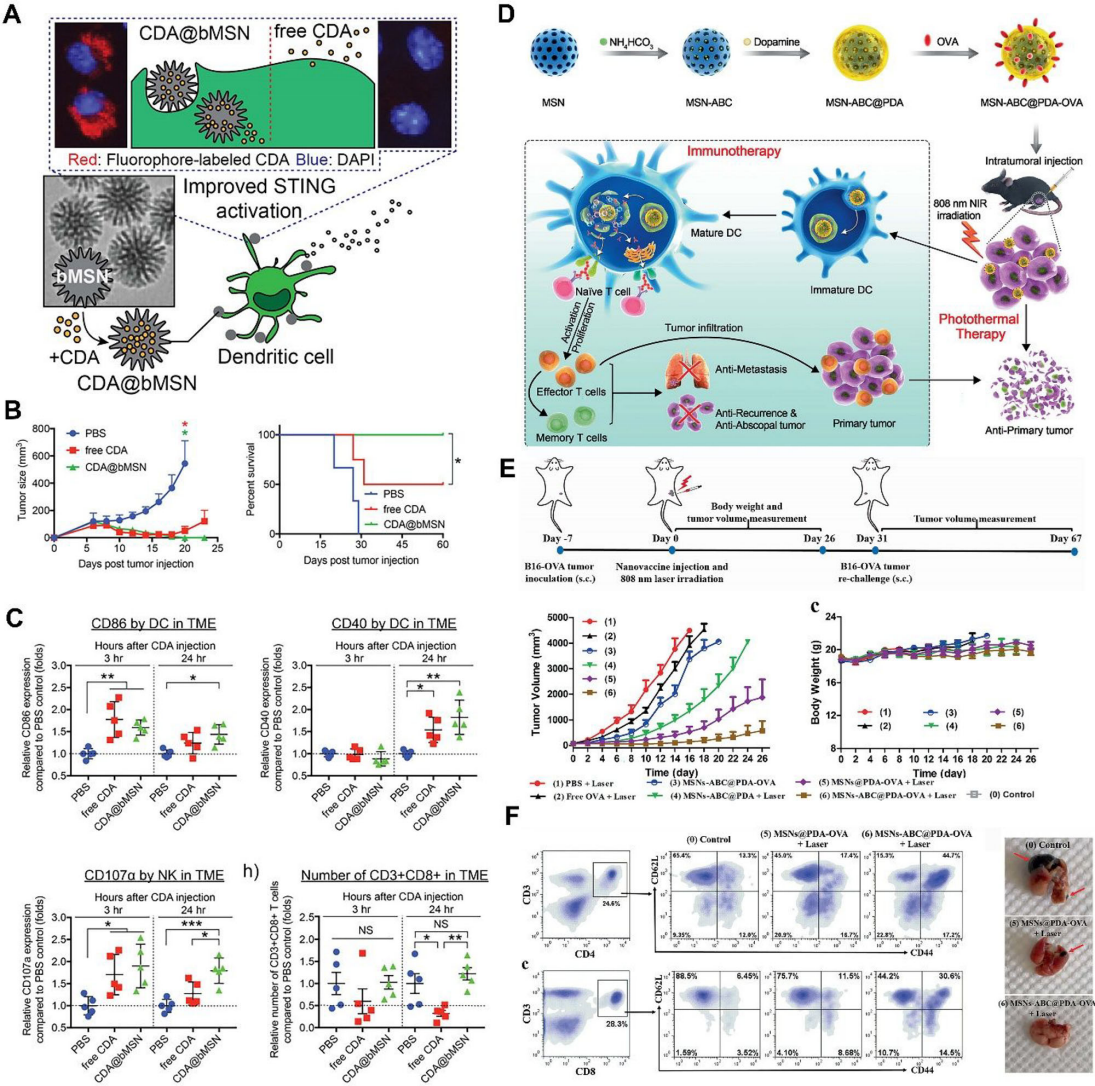
Mesoporous silica nanoparticles (MSN) for the delivery of antigen and adjuvants. Schematic illustration for the formulation of A) biodegradable MSN for STimulator of INterferon genes (STING) agonist delivery, B) tumor inhibition profiles and survival rates upon treatment, C) DC, natural killer cells, and T cell activation upon treatment. Reproduced with permission.^[^ ^88^ ^]^ Copyright 2020, Wiley. D) Schematic illustration for formulation of MSN coated with PDA and loaded with ovalbumin (OVA) for synergistic anti‐tumor immunotherapy. E,F) Tumor inhibition profiles and the T cell activation efficiency of nanoparticle treatment. Reproduced with permission.^[^ ^91^ ^]^ Copyright 2021, Wiley. CDA, *bis*‐(3′−5′)‐cyclic dimeric adenosine monophosphate; NK, natural killer.

MSN based cancer vaccines have been extensively studied, in most cases, model antigens such ovalbumin (OVA) or tumor antigens, or immune adjuvants (such as toll like receptor, TLR agonists) are loaded in MSN to provoke the immune response against cancer. Park et al. reported the delivery of a STimulator of INterferon genes (STING) agonist for effective cancer immunotherapy using biodegradable MSN (Figure 2A). In this report, MSN with a mean size of 80 nm and pore size of 5–10 nm were prepared with a thin wall which facilitated faster degradation of nanoparticles compared to conventional MSN. Single injection of STING agonist loaded MSN with a much lower dosage (< 5 μg) than typical dosage (10–240 μg) effectively activated DC and elicited potent innate and adaptive immune responses (Figure 2B,C).^[^ ^88^ ^]^ Nguyen et al. reported a 3D microporous scaffold composed of MSN coloaded with OVA and cytosine–guanine dinucleotide (CpG, a TLR9 agonist) for the activation of DC against cancer.^[^ ^89^ ^]^ Furthermore, Seth et al. studied the application of mesoporous silica core–shell structure for combined photothermal immunotherapy. The polydopamine core assisted the photothermal ablation of cancer whereas the release of TLR 7/8 agonist loaded in silica shell activated the immune response against cancer.^[^ ^90^ ^]^ Similarly, Huang et al. coated MSN with a PDA shell as a photothermal agent, then loaded OVA as the model antigen (Figure 2D). Under laser irradiation, the nanovaccine system realizes the release of antigens and induced robust anti‐tumor immune response (Figure 2E,F).^[^ ^91^ ^]^

Cheng et al. reported a biomimetic nanoplatform where the large pore dendritic MSN were loaded with CuS to induce photothermal ablation and resiquimod, an immune adjuvant. The composite was further coated with cancer cell membrane and finally conjugated with anti‐PD‐1 peptide. The peptides were released in the weak acidic TME of cancer, the immune adjuvant was released by the photothermal effect and hence resulted in potent tumor inhibition and T cell activation in the tumor.^[^ ^92^ ^]^ Wang et al. reported the use of metal‐doped MSN as cancer immunoadjuvants. In this study, Ca‐, Mg‐ and Zn‐doped MSN were prepared and their anti‐cancer immunity performance was assessed in vivo. Zn‐doped MSN when administrated with OVA exhibited potent T cell activation in vivo and Th1 cytokine secretion ex vivo.^[^ ^93^ ^]^

In addition to TLR agonists and model antigens, tumor antigens were also loaded in MSN to elicit immune responses. Liu et al. prepared a polyethyleneimine (PEI) modified thin shell hollow MSN (THMSN) for loading a melanoma derived peptide for inducing DC maturation, Th1 immunity, and immunological memory. THMSN vaccine demonstrated significant inhibition of tumor growth and metastasis with potent immune response.^[^ ^94^ ^]^ Our group recently reported the in situ phosphorylated TAA enrichment and delivery to immune cells using dendritic MSN (DMSN) system. DMSN was first coated with polydopamine and was further chelated with Ti^4+^ ions (TiDMSN). The nanoparticles with large surface area and high phosphoprotein enriching capability were used to enrich phosphorylated TAA and DAMPs released upon Dox treatment. When applied in combination with ICB therapy, TiDMSN exhibited potent tumor regression in both primary and secondary tumors. Also, enhanced infiltration of T cells as well as the maturation of DC and macrophages were observed in the distant tumor site of TiDMSN treated group.^[^ ^95^ ^]^

### 3.3 MSN for TME modulation

**FIGURE 3:**
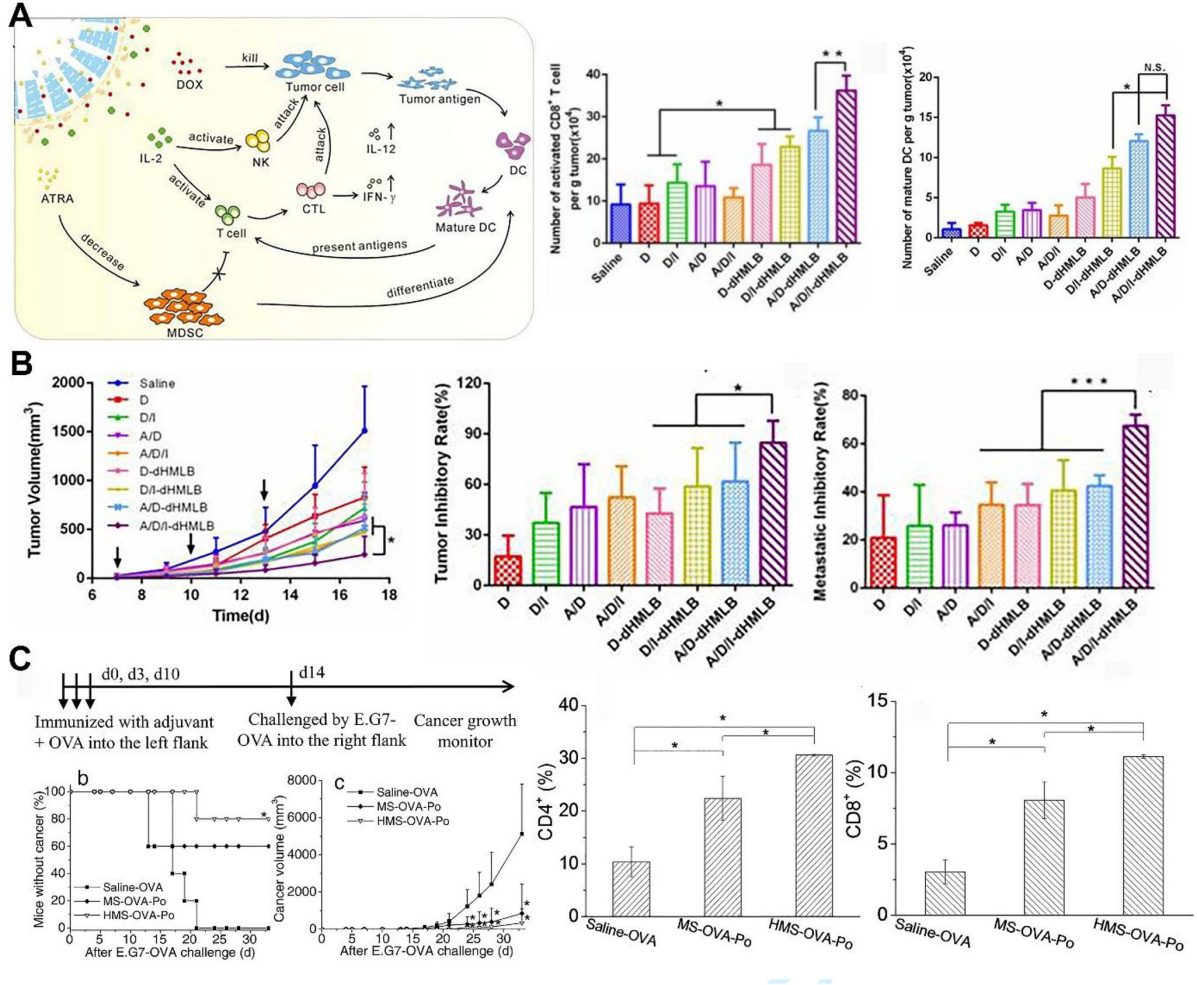
Mesoporous silica nanoparticles (MSN) for TME modulation and as a gene delivery vehicle. A) Schematic illustration of the induced synergistic antitumor efficacy of hollow MSN and the activation efficiency of CD8^+^ T cell and DC in the tumor. B) Tumor inhibition profiles, tumor inhibition rate, and metastatic inhibition rate upon different treatments. Reproduced under the terms of the Creative Commons Attribution license.^[^ ^96^ ^]^ Copyright 2017, Ivyspring International Publisher. C) Anti‐cancer and T cell activation efficiency of solid and hollow nanoformulations. Reproduced with permission.^[^ ^100^ ^]^ Copyright 2016, Wiley.

TME consists of various components that support tumor growth and evasion, hence the regulation of TME for effective cancer immunotherapy has emerged as a recent topic of interest. Kong et al. reported a combinational chemo‐immunotherapy strategy using a biodegradable hollow MSN loaded with all‐trans retinoic acid, Dox, and IL‐2. In this study all‐trans retinoic acid was used to differentiate myeloid‐derived suppressor cells (MDSC) into matured DC and thereby to reduce MDSC induced immunosuppression (Figure 3A,B). In addition, Dox is used as an ICD inducer and IL‐2 is used to facilitate T cell and natural killer cell activation in the tumor. This combinational nanosystem exhibited potent inhibition of tumor growth and metastasis.^[^ ^96^ ^]^ Furthermore, Yang et al. applied large pore DMSN modified with PEI and loaded with microRNA‐125a for the modulation of TME through polarization of tumor associated macrophages to anti‐tumor phenotype. Mice treated with the nano‐system exhibited significant reduction in tumor associated macrophages and MDSC in the tumor site as well as significant enhancement in tumor infiltrating T cells and natural killer cells.^[^ ^97^ ^]^

In another report, Jiang et al. reported a “nano catalytic medicine” composed of Fe^2+^‐Ru^2+^ loaded MSN to induce oxidative damage in mitochondrial DNA in tumor cells. The oxidative mitochondrial DNA acted as an immunogenic signal for macrophage polarization in the TME. These nanoparticles induced potent tumor inhibition in both primary and secondary tumors as well as the production of various inflammatory cytokines in blood serum.^[^ ^98^ ^]^ Furthermore, Li et al. reported a nanoenzyme based nanoformulation, where 5 nm CaO_2_ and Fe_3_O_4_ ultrasmall nanoparticles were loaded on DMSN and subsequently coated with a pH sensitive membrane for achieving tumor specific cargo release. In this system, both CaO_2_ and Fe_3_O_4_ facilitated Fenton like reaction for inducing ferroptosis in the tumor which helped in the modulation of TME and polarization of tumor associated macrophages.^[^ ^99^ ^]^

### 3.4 MSN as a gene delivery vehicle

In this section, the application of MSN as a gene delivery vehicle for cancer treatment is discussed in detail. As shown in Figure 3C), Wang et al. compared the anti‐cancer vaccination efficacy of solid and hollow mesoporous nanospheres loaded with OVA and Poly IC (a synthetic double‐stranded ribonucleic acid (dsRNA) analog). Mice immunized with hollow MSN showed enhanced anti‐tumor immunity with potent T cell activation against challenge due to enhanced cellular uptake by macrophage‐like cells.^[^ ^100^ ^]^ In another report, stellated fibrous mesoporous silica nanospheres were used for the delivery of Polyinosinic‐polycytidylic acid (Poly IC, a synthetic analog of double‐stranded RNA) which reduced the typical dosage of Poly IC required for inducing anti‐cancer immunity in vivo. Mice treated with the nanoparticle system exhibited comparable anti‐cancer immunity to the ones immunized with free Poly IC with 4 times dose.^[^ ^101^ ^]^

### 3.5 MON for drug‐mediated immunotherapy

**FIGURE 4:**
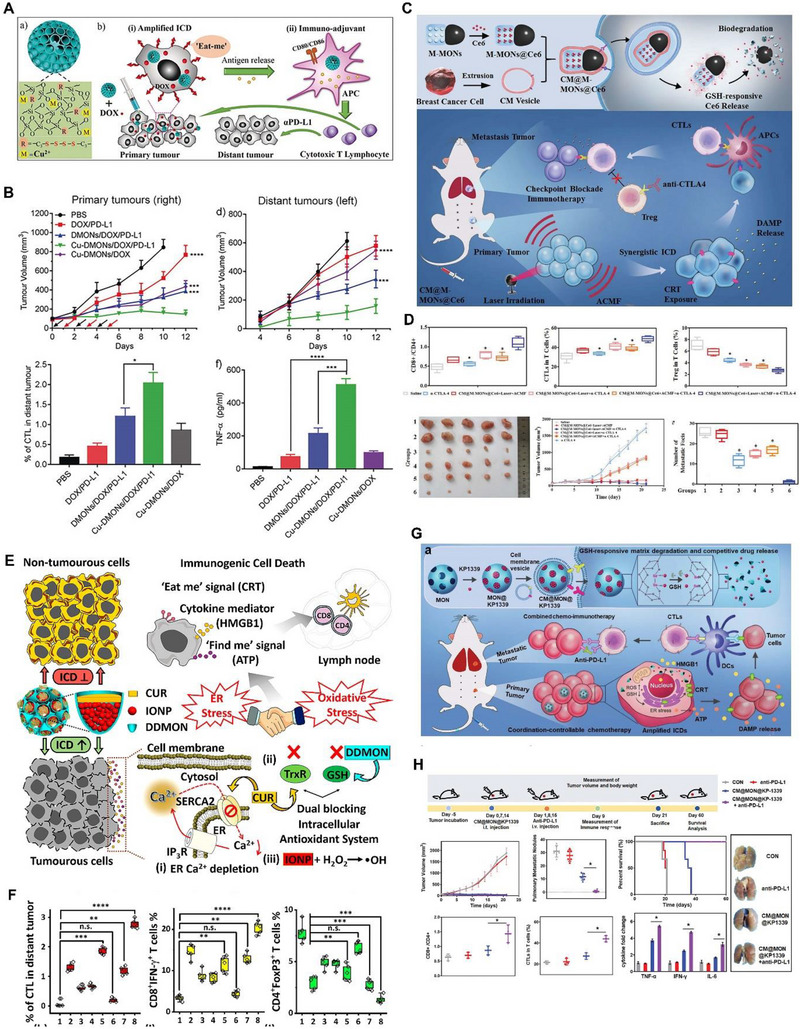
Mesoporous organosilica nanoparticles (MON) for drug delivery applications. Schematic illustration for the A) development of Cu^2+^ incorporated MON for boosting ICD in cancer and B) the anti‐tumor performance of the nanoparticle. Reproduced with permission.^[^ ^36^ ^]^ Copyright 2018, Wiley‐VCH. C) Schematic illustration of the formulation of Janus nanobullets for synergistic PDT and magnetic hyperthermia. D) Anti‐tumor performance and the T cell activation efficiency of the nanobullets. Reproduced with permission.^[^ ^102^ ^]^ Copyright 2019, Wiley‐VCH. E) Detailed mechanism of action of curcumin and iron oxide nanoparticle loaded MON for cancer immunotherapy and F) its T cell activation performance. Reproduced with permission.^[^ ^103^ ^]^ Copyright 2020, American Chemical Society. G) Synthesis and application of diselenide bridged MON for the delivery of KP1339. H) Tumor inhibition, T cell activation, and metastasis reduction upon treatment. Reproduced with permission.^[^ ^104^ ^]^ Copyright 2021, Wiley.

The possibility of tuning the framework composition of silica‐based nanoparticles opened a new arena of interest for the effective utilization of the nanocarriers as not just delivery vehicle but as biomodulators in biomedical applications. We reported the cupper ions incorporated tetrasulfide bridged dendritic MON (DMON) for chemo‐immunotherapy for treating breast cancer. This design utilized the Fenton agent Cu^2+^ to produce reactive oxygen species (ROS) and tetrasulfide organic groups to deplete intracellular antioxidant GSH, resulting in enhanced intracellular oxidative stress and improved Dox mediated ICD (Figure 4A). The Cu incorporated organosilica nanoreactors exhibited synergistic tumor inhibition and immune response when applied in combination with ICB therapy (Figure 4B).^[^ ^36^ ^]^ Wang et al. prepared Janus mesoporous organosilica nanobullets for synergistic anti‐metastatic immunotherapy (Figure 4C). The nanobullets consist of a magnetic head compartment and Chlorin e6 (Ce6, a photosensitizer) loaded disulfide bridged mesoporous organosilica body compartment. The nanobullets were subsequently coated with a cancer cell membrane to assist tumor targeting and immune escape. The GSH responsive nanobullets effectively released Ce6 in tumor to achieve a synergistic therapeutic outcome from PDT and magnetic hyperthermia. Janus magnetic MON when applied in combination with ICB therapy elicited potent suppression of metastatic tumors (Figure 4D).^[^ ^102^ ^]^

Dai et al. reported a tumor specific organosilica based nanoinducer of ICD (Figure 4E,F). Disulfide bridged organosilica nanoparticles were co‐loaded with curcumin and iron oxide nanoparticles which can deplete intracellular GSH, generate hydroxyl radicals, induce calcium depletion, and thioredoxin reductase inhibition. The highlight of this work is that the individual components of the nanoplatform are unable to trigger ICD; however, when combined the synergistic biological activities together, potent anti‐tumor immune response was triggered. While GSH depletion and delivery of iron oxide nanoparticles can induce oxidative stress, curcumin induces endoplasmic reticulum stress by interfering the calcium homeostasis in cells. The nanoformulation successfully induced ICD as characterized by the enhanced expression of calreticulin, release of HMGB1 and ATP in cancer cells. Moreover, enhanced maturation of DC in lymph nodes and infiltration of cytotoxic T lymphocytes (CTL) in distant tumors were also demonstrated.^[^ ^103^ ^]^

Chen et al. reported the application of large pore MON loaded with OVA and iron oxide core (IO‐LPMON‐OVA) for the simultaneous activation of T cells and polarization of TAM. In this work the nanoformulation exhibited potent activation of CD4, CD8 T cells and polarization of macrophages in vitro as well as in vivo. Mice immunized with IO‐LPMON‐OVA significantly restrained tumor growth after tumor inoculation.^[^ ^37^ ^]^ Shao et al. reported the diselenide bridged MON responsive to X‐ray and ROS for controlled delivery of Dox. The nanoplatform was further coated with cancer cell membrane and applied in combination with ICB therapy for enhanced inhibition of tumor and metastasis. Diselenide bridging group has a similar functional characteristic to disulfide bridging group which degrades in the presence of GSH, providing GSH depletion function in cells. In this report, it was further elucidated that X‐ray radiation sensitive diselenide bond can be also used for the X‐ray radiation responsive release of drug. This study achieved a controlled release of Dox with low radiation dose due to the dual responsive nature of diselenide bonds (i.e., X‐ray and GSH).^[^ ^32^ ^]^ Similarly, as shown in Figure 4G, Zhang et al. reported an ICD nanoamplifier containing diselenide bridged MON for the delivery of a chemotherapeutic ruthenium compound (KP1339 an ICD inducer). In addition to the GSH depleting nature of diselenide groups, selenium can be also coordinated with metals such as rubidium for the effective delivery of metal‐based drugs. The nanoparticles are further coated with cancer cell membrane and applied in combination with ICB therapy. The organosilica nanoformulation induces the generation of ROS and depletes GSH for evoking enhanced ER stress which amplifies ICD by the rubidium‐based drug. Compared to nanoparticle or ICB therapy alone, when used in combination the nanoformulation exhibited potent tumor inhibition, T cell activation, and reduced lung metastasis (Figure 4H).^[^ ^104^ ^]^ In another report, Yang et al. also reported the synthesis of diselenide bridged MON coloaded with Dox and methylene blue for chemo‐PDT at a low red light radiation dosage. The nanoformulation exhibited boosted chemo‐PDT mediated ICD and produced robust anti‐tumor immune response with enhanced T cell infiltration in the distant tumor site.^[^ ^105^ ^]^

### 3.6 MON for the delivery of antigens and adjuvants

**FIGURE 5:**
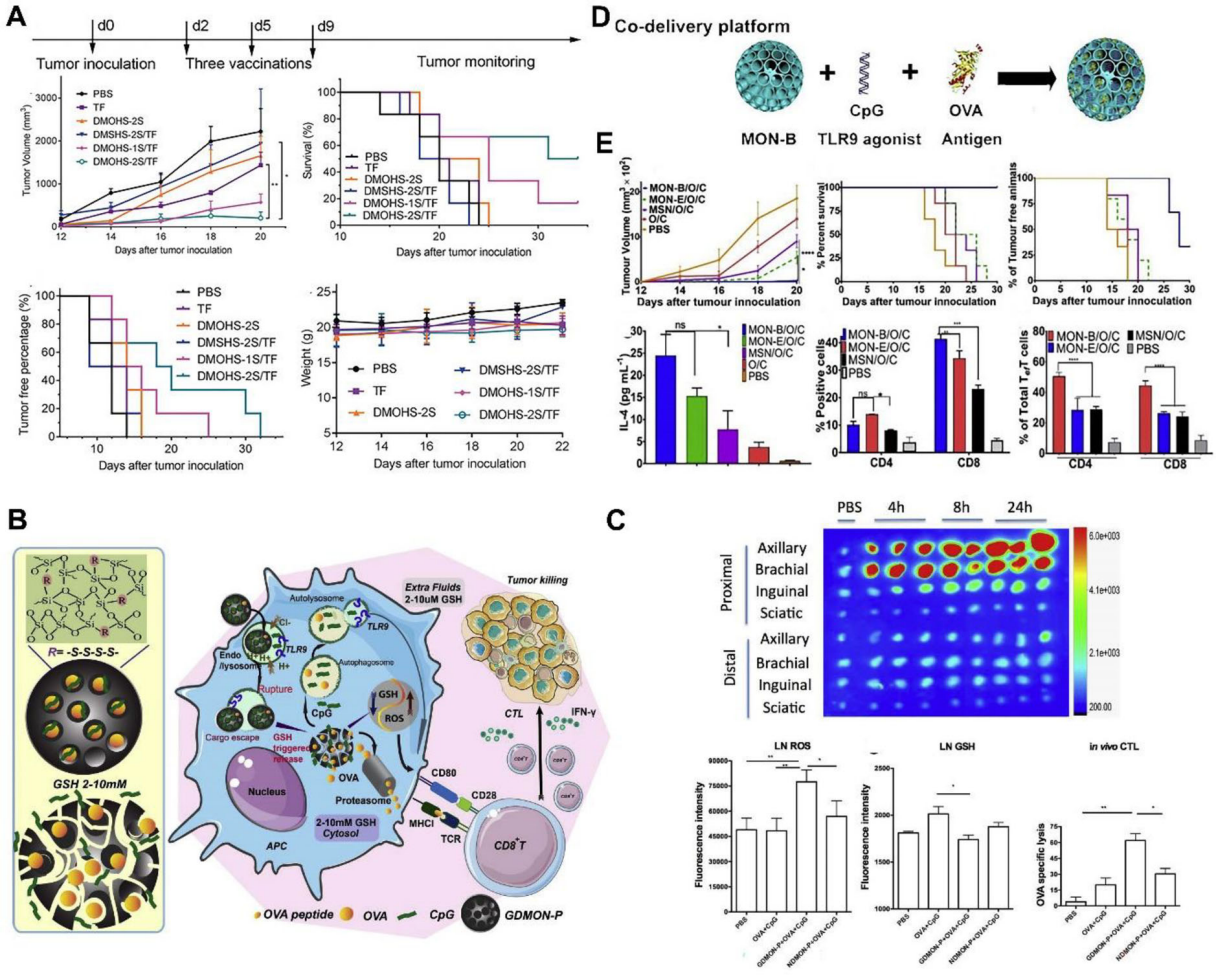
Mesoporous organosilica nanoparticles (MON) for the delivery of antigens and adjuvants. A) Anti‐tumor performance of hollow MON with adjustable number of shells. Reproduced with permission.^[^ ^106^ ^]^ Copyright 2017, Wiley‐VCH. B) Scheme for the development of biodegradable MON for the delivery of ovalbumin (OVA) and CPG. C) Accumulation of nanoparticles in the lymph nodes over time, ROS and GSH levels in lymph nodes, and CTL activation efficiency. Reproduced with permission.^[^ ^107^ ^]^ Copyright 2018, Elsevier. D) Schematic illustration of the loading of CpG and OVA in benzene bridged MON and E) its anti‐tumor performance. Reproduced with permission.^[^ ^108^ ^]^ Copyright 2020, Elsevier.

Our group reported the first DMON hollow spheres with adjustable number of shells as an adjuvant in cancer immunotherapy (Figure 5A). The nanoparticles were adjusted to have single and double shells where the double shell structure exhibited better sustained antigen release and enhanced immune response in vivo.^[^ ^106^ ^]^ In another report, we demonstrated the application of GSH responsive biodegradable tetrasulfide bridged DMON for effective co‐delivery of OVA and CpG in antigen presenting cells such as macrophages for initiating immune response against cancer (Figure 5B,C).^[^ ^107^ ^]^ Our group recently demonstrated the application of benzene bridged DMON as an effective immunoadjuvant for the co‐delivery of OVA and CpG (Figure 5D). This study compared the influence of different framework compositions such as inorganic silica, ethylene bridged, and benzene bridged silica on their cancer immunotherapeutic performance. It is shown that the benzene bridged composition with high hydrophobicity can significantly stimulate the activation of immune cells (Figure 5E). Mice treated with benzene bridged MON coloaded with OVA and CpG exhibited potent tumor inhibition and activation of T cells in the splenocytes.^[^ ^108^ ^]^

### 3.7 MON for TME modulation

**FIGURE 6:**
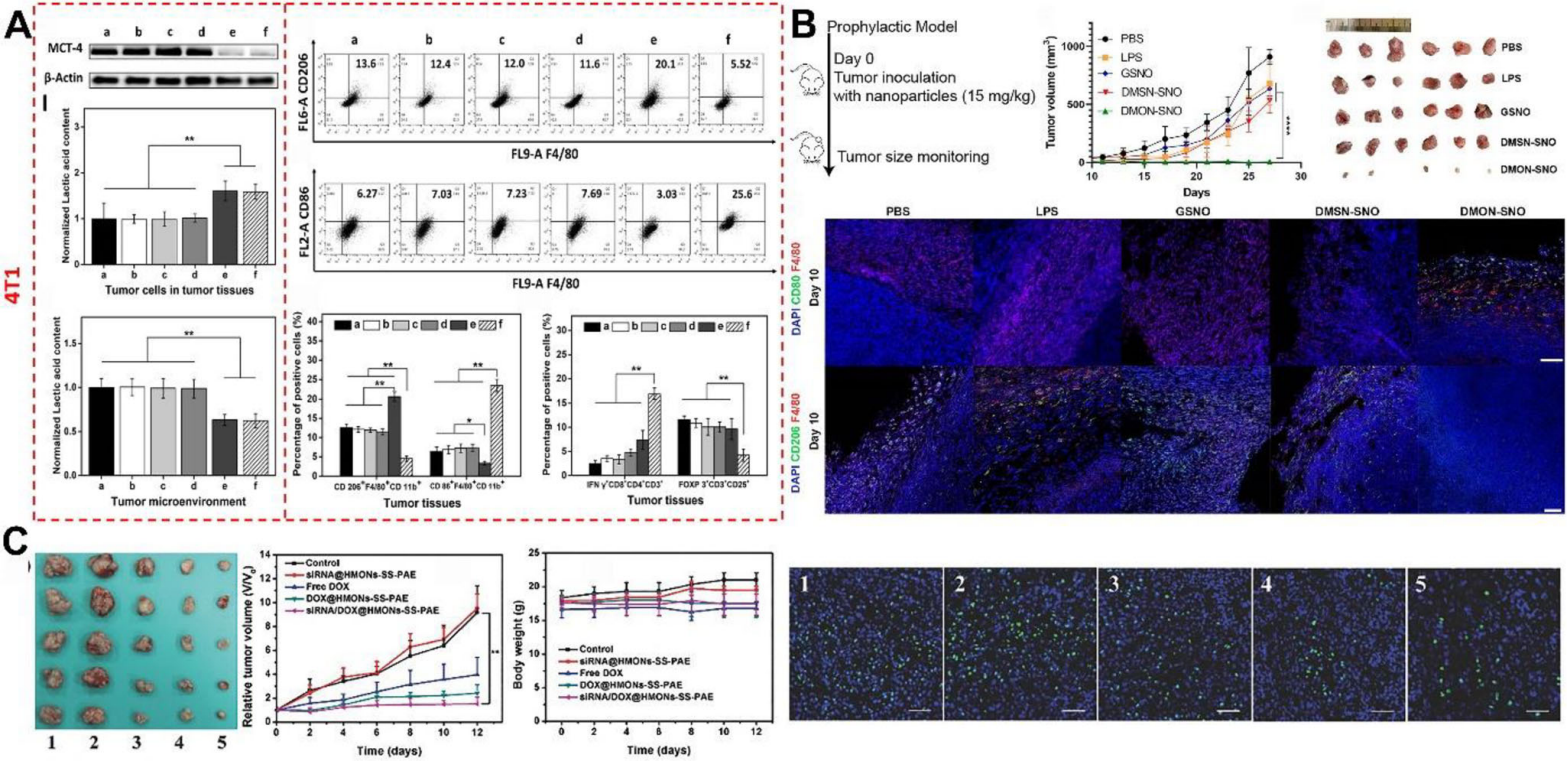
Mesoporous organosilica nanoparticles (MON) for TME modulation and as gene delivery vehicle. A) Lactic acid normalization efficiency, macrophage polarization, and T cell activation by different nanoparticle treatments (a: Saline, b: HMONs‐BSA‐PEI‐CDM‐PEG, c: HCPT, d: HMONs@HCPT‐BSA‐PEI‐CDM‐PEG@siNC, e: HMONs@HCPT‐BSA‐PEI‐ CDM‐PEG@siMCT‐4 + forskolin, f: HMONs@HCPT‐BSA‐PEI‐CDM‐PEG@siMCT‐4). Reproduced with permission.^[^ ^109^ ^]^ Copyright 2020, American Chemical Society. B) Tumor inhibition efficiency and macrophage polarization by nitric oxide delivering MON. Reproduced with permission.^[^ ^110^ ^]^ Copyright 2022, American Chemical Society. C) Anti‐tumor efficiency of gene loaded MON compared to other treatments and the Ki67 protein expression in tumor sections upon different treatments (1: control; 2: siRNA@HMONs‐ss‐PAE; 3: free DOX; 4: DOX@HMONs‐ss‐PAE; 5: siRNA/DOX@HMONs‐ss‐PAE). Reproduced with permission.^[^ ^111^ ^]^ Copyright 2016, Wiley.

In recent years, TME modulation and reversal of immunosuppressive TME are attractive topics in cancer immunotherapy. Li et al. formulated a nanoplatform that is responsive to weak acid in TME and high GSH in cancer cells in a cascade manner. Hollow MON were prepared and loaded with hydroxycamptothecin (HCPT) and siMCT‐4 to trigger the inhibition of lactate efflux from cells. Subsequently, accumulation of intracellular lactate level induced cell apoptosis as well as reduced lactate level in extracellular environment induced polarization of TAM which improved the CD8^+^ T cell activity by effectively regulating immunosuppressive TME. Macrophage polarization, lactate acid normalization in TME, improved T cell activity, enhanced tumor inhibition, and reduced lung metastasis were demonstrated in both B16F10 and 4T1 tumor models (Figure 6A).^[^ ^109^ ^]^ We recently reported s‐nitrosothiol modified DMON based nitric oxide donors for the reversal of immunosuppressive TME through activation and polarization of macrophages to their anti‐tumor phenotype. It is demonstrated that the GSH depleting capability of tetrasulfide bridged organic groups can be utilized to enhance the intracellular nitric oxide delivery in macrophages. The activation of macrophages through effective intracellular nitric oxide delivery has been demonstrated in immortalized RAW 264.7 cell lines, bone marrow derived macrophages as well as in human monocytes. The nanoparticles exhibited enhanced tumor inhibition and activation of macrophages in the tumor site following both prophylactic and therapeutic tumor models (Figure 6B).^[^ ^110^ ^]^

### 3.8 MON for gene delivery

In this section, the reports on the use of MON for gene delivery mediated cancer immunotherapy are summarized. Wu et al. prepared hollow MON with disulfide bridging group and capped with poly (β‐amino esters) (PAE), then co‐loaded a P‐glycoprotein modulator siRNA to reverse multi drug resistance and Dox to induce anti‐cancer effect (Figure 6C). Disulfide bridging groups and PAE were used for the GSH responsive drug release, while PAE was also utilized as a polycation with ester bonds for loading and releasing siRNA intracellularly.^[^ ^111^ ^]^ Similarly, Sun et al. reported a core/shell silica/organosilica nanosystem with small and large pores for the independent loading of large siRNA molecules and small Dox molecules. It is demonstrated that the organosilica shell loaded with siRNA will degrade in tumor, releasing the gene molecules first followed by the release of Dox. The released siRNA in the first stage reversed the multi‐drug resistance in cancer cells while the subsequently released Dox induced anti‐tumor effect.^[^ ^112^ ^]^

Although, as discussed above, different types of MSN and MON have been prepared with precise control of structural, compositional, and morphological aspects to suit the target biomedical applications, it is important to note that the suitability of silica based nanoplatform as a nanomedicine for cancer immunotherapy also depends on the biocompatibility of these nano systems in physiological conditions. Hence, the biosafety assessment of MSN and MON will be briefly reviewed in the next section.

## 4 BIOSAFETY ASSESSMENT OF MSN AND MON

In vivo toxicology of nanoparticles is a long‐term hurdle in the effective utilization of nanoparticles in the clinical settings. Even though MSN have been used in the field of drug delivery for more than two decades, it has been only approved as a food additive and as an excipient in oral drug formulations.^[^ ^113^ ^]^ The in vivo biocompatibility of silica based nanoparticles depends on various factors including composition, degradability, size, shape, surface modification, the route of administration, etc.^[^ ^114^ ^]^ However, a comprehensive understanding on the interplay and influence of all these factors on biosafety is still lacking. It is also difficult to directly compare the biocompatibility of different types of silica‐based nanoparticles, because usually 1) there are more than one structural parameters changed in a group of samples under investigation, 2) the batch‐to‐batch and lab‐to‐lab variations in the synthesis of samples with quality control; and 3) the dosage of administration in different studies may vary. Nevertheless, there are some general trends that one can follow.

Inorganic silica nanoparticles tend to accumulate in liver, spleen, and lungs upon 24 h of intravenous injection.^[^ ^113^ ^]^ It has also been found that polyethylene glycol modification of MSN can improve the bioavailability and reduce the capture by liver, spleen, and lungs.^[^ ^115^ ^]^ Nevertheless, the presence of rigid framework which leads to the slow degradation and clearance of MSN is a concern on biosafety. Cho et al. studied the size impact of MSN on the distribution and elimination of MSN after single intravenous injection. In this study, the clearance of silica nanoparticles of three sizes (50, 100, 200 nm) was tested where the smallest size got cleared faster than the larger sizes through urine and bile. The larger nanoparticles were trapped in macrophages in spleen and liver, and remained in site for 4 weeks after injection.^[^ ^116^ ^]^ The MSN are known to undergo in vivo degradation, the rate of which depends on the available surface area as well as any chemical modifications done on the surface.^[^ ^114^, ^117^ ^]^ Therefore, the degradation occurs faster in nanoparticles with high specific surface area.^[^ ^118^, ^119^ ^]^ Furthermore, silica nanoparticle degradation can be accelerated through PEI modification as tested in acidic and physiological pH (5.0 and 7.4, 37°C), leading to 81% degradation within a week relative to 68% in unmodified MSN.^[^ ^120^ ^]^ PEGlyation of silica nanoparticle surface is commonly used to increase in vivo half‐life and reduce immunogenic response.^[^ ^121^ ^]^ However, PEGylation may also result in delayed degradation capability of nanoparticles,^[^ ^122^ ^]^ which may lead to accumulation in excretion organs due to prolonged time of exposure and lack of degradation.

When compared to MSN, the biodegradable nature of MON can be improved by controlling their framework composition to make them more biocompatible. The bridging groups such as disulfide and diselenide are stable in physiological conditions such as in blood but starts to degrade upon interacting with thiols, for example in tumor.^[^ ^114^ ^]^ However, the in vivo clearance and toxicology of these degraded products were rarely studied possibly due to the difficulties in the accurate characterization of complete degradation and clearance dynamics in the in vivo conditions. On the other hand, dissolution rates of the nanoparticles can be also slowed down by incorporating bridging groups such as ethylene and phenylene.^[^ ^54^, ^123^ ^]^ Therefore, a detailed and comprehensive understanding of the degradation profiles, elimination pathways of degraded products, and the time taken for the removal is required for successful application of silica‐based nanoparticles in nanomedicine.

## 5 OUTLOOK AND CHALLENGES

Both MSN and MON are attractive nanosystems for cancer research especially for cancer immunotherapy due to their large specific surface area, adjustable pore size, high pore volume, ease in performing surface modifications, and a variety of framework compositions. Even though, MSN were applied in cancer immunotherapy mainly as a versatile drug carrier, while the next generation MON have gained increasing interest because MON can be applied as not just nanocarriers but also biomodulators to boost the performance of the therapeutic molecules delivered. In this review article we have summarized the literature on the use of MSN and MON in different fields of cancer immunotherapy. However, the application of MON in cancer immunology is still in its infancy. Successful application of silica based nanosystems for nanomedicine requires further in‐depth investigations, such as in the following directions.

1. The fundamental synthesis mechanisms for both MSN and MON are similar, however, the rates of hydrolysis and condensation of different organosilane precursors vary greatly and their self‐assembling process with surfactants can also vary depending on the precursors used and reaction conditions. This makes the synthesis and structural control of different framework compositions difficult. This would in turn make the large‐scale production and quality control of these materials difficult, but this is a major task for future applications.
2. The current trend in applying silica based nanosystems in conjunction with other immunotherapeutic strategies such as chemotherapy, PTT, PDT, ICB has resulted in enhanced performance in terms of therapeutic outcome. However, the applications of MON with intrinsic physiochemical properties different from MSN in cancer immunotherapy are still limited. The anomaly in cancer and its TME can be potentially manipulated through the design of appropriate silica‐based nano‐platforms, specifically MON, to gain ultimate utilization of nanoparticles as well as to reduce the toxicity of other immunotherapeutic strategies mentioned above.
3. Although there are numerous reports on the application of MSN for cancer immunotherapy, it is still not clear whether these nanoparticles have any intrinsic immune modulating properties themselves. Also, there have been few direct comparisons between MSN and MON on such properties. Understanding the functionality difference between MSN and MON may assist in the rational design of silica‐based nano‐systems with elicited immune responses in cancer immunotherapy applications.
4. When considering the application of MON in cancer immunotherapy, the investigations on the application of different framework compositions are still limited. GSH responsive degradability of MON using tetrasulfide or disulfide or diselenide bridging groups is heavily reported. Apart from this, the application of benzene bridged organosilica nanoparticles as an adjuvant in cancer immunotherapy has been reported. However, still there is a gap in the utilization of other framework compositions which can synergistically enhance the immunotherapeutic performances. Moreover, it is also important to systematically investigate the biosafety of various compositions in addition to their therapeutic advantages. For instance, when the organic compositions such as ethylene or phenylene are used, the stability of the bridges would have significant impact on the bio‐degradability and biosafety of the nanoparticles.
5. Maintaining the porous nature and uniform morphology of MON while reducing the size of nanoparticles is one of the major challenges in the successful translation of these nanoparticles in therapeutic applications where i.v administration is predominantly applied. These nanoplatforms can also be appropriately engineered to suit other non‐invasive administration systemic drug delivery routes such as oral to uncover their immune regulating properties against cancer.
6. Previous reports on the use of MSN and MON sometimes include a biocompatibility assessment, accumulation profiles in different organs, degradability over time in vitro settings, etc. However, it is still not lucid whether these nanoparticles can be completely removed from the body, if yes, how long does it take and through which mechanism? Does the accumulation raise any long‐term biosafety concerns? Moreover, the biosafety assessments in previous reports are not directly comparable due to the differences in synthetic procedures, structures, organosilica contents, and treatment procedures. This makes the therapeutic translation of these candidates or any silica based nanoplatforms difficult.

In conclusion, based on the research progresses presented in this review, silica‐based nano‐platforms, both silica, and organosilica in composition, can be potential tools for nanomedicine mediated cancer immunotherapy. Although, great efforts have been rendered in this field, there are still many unanswered questions; and the true potential of these nanoparticles has not been fully uncovered. Due to the bottlenecks present in the application of MSN, nano‐therapeutics using MON can be further explored by paying special attention to the physiochemical properties, composition, and designs focusing on the targeted branches of cancer immunotherapy for future nanomedicine and clinical translation.

## CONFLICT OF INTEREST STATEMENT

The authors declare no conflicts of interest.

## FUNDING INFORMATION

Australian Research Council, Queensland Government, the University of Queensland and the Queensland node of the Australian National Fabrication Facility
